# Sociodemographic and reproductive risk factors associated with metabolic syndrome in a population of Brazilian women from the city of Ribeirão Preto: a cross-sectional study

**DOI:** 10.61622/rbgo/2024AO08

**Published:** 2024-03-15

**Authors:** Ênio Luis Damaso, Heloisa Bettiol, Viviane Cunha Cardoso, Mariane Nunes de Nadai, Elaine Christine Dantas Moisés, Carolina Sales Vieira, Ricardo Carvalho Cavalli

**Affiliations:** 1 Universidade de São Paulo Bauru Dental School Department of Pediatric Dentistry, Orthodontics and Public Health Bauru SP Brazil Department of Pediatric Dentistry, Orthodontics and Public Health, Bauru Dental School, Universidade de São Paulo, Bauru, SP, Brazil.; 2 Universidade de São Paulo Faculdade de Medicina de Ribeirão Preto Ribeirão Preto SP Brazil Faculdade de Medicina de Ribeirão Preto, Universidade de São Paulo, Ribeirão Preto, SP, Brazil.

**Keywords:** Obesity, Metabolic syndrome, Cohort study, Risk factors

## Abstract

**Objective:**

To identify sociodemographic and reproductive risk factors associated with MetS in women in their fourth decade of life.

**Methods:**

Cohort study conducted on women born from June 1978 to May 1979 in Ribeirão Preto, Brazil. Sociodemographic, clinical, and obstetric data were collected by interview and clinical evaluation. Univariable and multivariable binomial logistic regression models were constructed to identify the risk factors of metabolic syndrome and the adjusted relative risk (RR) was calculated.

**Results:**

The cohort included 916 women, and 286 (31.2%) of them have metabolic syndrome. MetS was associated with lack of paid work (RR 1.49; 95% CI 1.14-1.95), marital status of without a partner (RR 1.33; 95% CI 1.03-1.72), low educational level (less than 8 years of schooling [RR 1.72; 95% CI 1.23-2.41], 8 to 12 years of schooling [RR 1.37; 95% CI 1.06-1.76], when compared with more than 12 years of schooling), and teenage pregnancy (RR 2.00; 95% CI 1.45-2.77). There was no association between MetS, and the other covariates studied.

**Conclusion:**

Metabolic syndrome in a population of women in the fourth decade of life was associated with lack of employment, lack of a partner, low educational level, and teenage pregnancy.

## Introduction

Metabolic syndrome (MetS) is a complex of interrelated risk factors for cardiovascular disease (CVD). These factors include obesity (especially central obesity), dysglycemia, arterial hypertension, and dyslipidemia (elevated triglyceride levels and low high-density lipoprotein cholesterol levels).^(1)^

The first diagnostic criterion for MetS was proposed by the World Health Organization (WHO), in 1999.^(2)^ In the same year, the European Group for the Study of Insulin Resistance (EGIR) proposed a new definition.^(3)^ In 2001, in the United States, the National Cholesterol Education Program Adult Treatment Panel III (NCEP-ATP III) proposed a new definition in which the diagnosis of MetS includes the co-occurrence of at least three of the five components mentioned.^(4)^ In 2005, the American Heart Association and the National Heart Lung and Blood Institute (AHA /NHLBI) changed only the fasting blood glucose cutoff from 110 to 100 mg/dl in a review of these criteria after the American Diabetes Association (ADA) suggested adjustments.^(5)^ As recently as 2005, the International Diabetes Federation (IDF) proposed standardization of the existing diagnostic criteria to take into account the presence of mandatory central obesity, measured by waist circumference, along with the presence of two other risk factors.^(6)^

In 2015, the Brazilian Society of Cardiology proposed NCEP-ATP III criteria suitable for the diagnosis of MetS.^(7)^

In general, all MetS diagnostic criteria consider the presence of dyslipidemia (elevated triglyceride levels and low high-density lipoprotein cholesterol levels), arterial hypertension, obesity, and hyperglycemia. Mandatory criteria for analysis, as well as different reference values for arterial hypertension and other biochemical measurements, have been proposed, with no consensus on which combination of risk factors should be considered in the final diagnostic criterion for MetS.^(8)^

The incidence of metabolic syndrome often parallels the incidence of obesity and incidence of type 2 diabetes mellitus (T2DM). There is no global data on metabolic syndrome—which is harder to measure, but since MetS is about three times more common than diabetes, the global prevalence can be estimated to be about one quarter of the world population.^(9)^ In Brazil, the prevalence of MetS in the adult population was found to be 29.6%,^(10)^ reaching more than 40% in age groups over 60 years old.^(11)^

Because MetS is associated with excess body fat, it has risk factors similar to overweight and obesity and their clinical and metabolic consequences. The following conditions have been described as risk factors for the development of MetS: positive family history, smoking, increasing age, obesity, low socioeconomic status, menopause, sedentary lifestyle, high sugar consumption, and excessive alcohol consumption.^(12,13)^

The prevalence of MetS in women ranges from 10.7% to 40.5%, depending on the population studied and diagnostic criteria, and may be associated with various women’s diseases such as polycystic ovary syndrome and gestational diabetes.^(7)^

The prevalence of MetS shows a progressive increase between the premenopausal and postmenopausal periods, with ethnic heterogeneity, age, socioeconomic factors, lifestyle, age at menarche, and number of pregnancies noted as possible factors that may influence the increase in prevalence in women.^(14)^ These data from previous literature suggest that obstetric history and possibly pregnancy may influence the development of obesity and chronic diseases associated with MetS.

Metabolic syndrome and obesity are diseases of great concern in both women and men, as their prevalence is increasing, and the risk of cardiovascular disease and mortality is rising. Since Brazil is a very large country, it is important to focus on smaller geographic areas and individuals, since they have different levels of development and the predictors of prevalence of chronic diseases and MetS could play a different role. Epidemiological knowledge enables the development of public policies to prevent and promote healthy habits in a society.

Thus, the primary objective of the present study was to identify sociodemographic and reproductive risk factors associated with metabolic syndrome in women in the fourth decade of life, using a birth cohort analyzed since 1978/79. The secondary objectives were to identify reproductive risk factors for metabolic syndrome in women with previous pregnancies and to describe the prevalence of chronic diseases (obesity, diabetes mellitus, systemic arterial hypertension, dyslipidemia, and metabolic syndrome) in women in the fourth decade of life.

## Methods

This was a cross-sectional observational study embedded in a cohort study. The sample of this study consisted of women from the 1978/79 birth cohort, conducted in the city of Ribeirão Preto, São Paulo State, Brazil.^(15)^ This is the first Brazilian cohort that evaluated these data.

Ribeirão Preto is a Brazilian city in the state of São Paulo, a rich and industrialized region with a Human Development Index (HDI) of 0.800, and a population of 698,259 in 2022.^(16)^ It is one of the most developed cities in the country, with 99% of homes having running water and a sewage system.^(16)^

**Figure 1:**
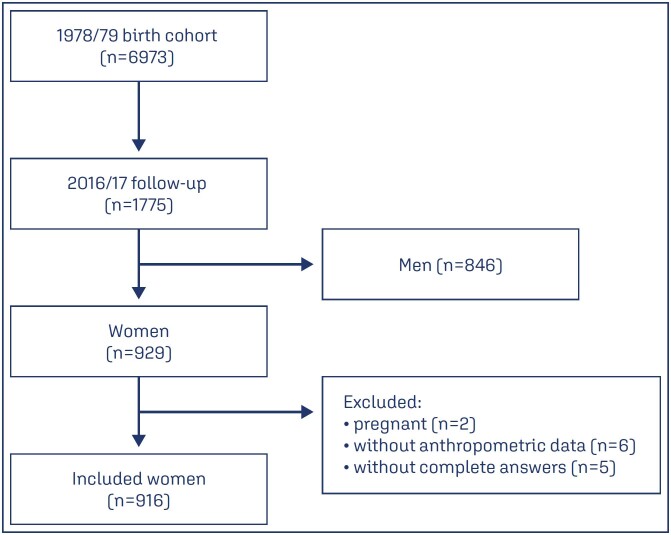
Flow chart of participants in the 1978/79 birth cohort in Ribeirão Preto

At the beginning of the cohort study in the late 1970s, the records and charts of three public and five private maternity hospitals were evaluated, in which 98% of all deliveries in the community^(15)^ from June 1978 to May 1979 (n = 6,973) were reviewed.^(17)^ In the following two years, the city’s registry offices were visited to record the deaths in the first year of life of the children born during this period.^(15)^ The first follow-up of this cohort occurred in 1987/1989, when the children were sought in schools;^(18)^ 2,898 children aged 8 to 11 years were evaluated.^(19)^ The second follow-up occurred in 1996/1997, when 2,083 male participants aged 18 or 19 years were evaluated on the occasion of enlistment in military service.^(18)^ Between 2002 and 2004, the cohort was again visited and 2,103 participants aged 23 to 25 years were assessed.^(15)^ The last follow-up of this cohort took place in 2016/2017, when 1,775 participants (25% of the original sample) aged 37 to 39 years were evaluated (Figure 1).

The mothers interviewed, from whom data and data on their children were collected, gave verbal consent and were discharged by their responsible physicians.^(15)^ The last follow-up of the study was submitted to the Research Ethics Committee of the University Hospital, Ribeirão Preto Medical School, University of São Paulo and was approved under number 1.282.710.

Women who had participated in the last follow-up (2016/17) were included in the present study (n=929). Participants were invited to come to the research center on a specific day and time. Eligible participants were informed of the aims of the study and asked to sign the free informed consent form. Data collection did not begin until participants signed the form.

Women who were pregnant at the time of the study (n=2), women who did not undergo anthropometric data collection (n=6), and women who did not answer the obstetric history questionnaire (n=5) were excluded. Pregnant women were excluded because anthropometric data may not reflect a diagnosis of obesity and metabolic syndrome, depending on gestational age.

Questionnaires containing demographic, social, clinical, and reproductive data were used in the surveys (2016/17).

The following sociodemographic variables were collected: race/ethnicity (white and others), paid work (to be engaged in a paid activity at the time of application of the questionnaire), socioeconomic class, stable marital status (to live with a partner [married, cohabiting or stable relationship] or not [single, widowed]), educational level (years of schooling: ≤ 8 years, 9 – 11 years, ≥ 12 years), smoking (it was considered present if the patient answered that she had the habit of smoking, regardless of the number of cigarettes), alcohol misuse (alcohol abuse was considered to be the consumption of more than 3 doses in a day or more than 7 doses in a week, each dose being the equivalent of 10 g of alcohol),^(20)^ and illicit drug use (the habit of using any illicit drug [cocaine, marijuana, opiates, volatile solvents and hallucinogens] was marked as present, regardless of the frequency of use).

Socioeconomic status was defined according to the Associação Brasileira de Empresas de Pesquisa (ABEP)^(21)^ categories. The estimated monthly household income for each ABEP category was: A (>20× minimum wage), B (10–20× minimum wage), C (4–10× minimum wage), D (2–4× minimum wage), and E (<2× minimum wage).^(21)^ The minimum wage in Brazil at completion of this study was US$267.81 per month.

The clinical variables used in this study were those related to the diagnosis of metabolic syndrome: diabetes mellitus, dyslipidemia, obesity, and arterial hypertension. The presence of these comorbidities was considered positive if the woman was already aware of this diagnosis, if she was taking medication for these diseases, or if the diagnosis was made during the examination by physical examination or laboratory tests.

Variables related to sexual and reproductive health included previous pregnancies, number of previous pregnancies (0, 1, and ≥ 2), age at first pregnancy (< 20 years, 20 to 29 years, > 30 years), previous abortion, parity (0, 1, and ≥ 2), previous cesarean section, and breastfeeding (breastfeeding more than half of children or less).

Participants underwent body composition assessment, anthropometry, blood sampling, and blood pressure measurement. Trained health personnel collected the data. Information on chronic diseases was collected by interview. An existing medical condition was present if the participant was diagnosed with systemic arterial hypertension, diabetes mellitus, dyslipidemia, or was receiving treatment.

Body height was measured with a stadiometer attached to a smooth wall, scaled in centimeters, with the patient standing upright and barefoot, arms extended along the body, head in the Frankfurt plane (imaginary line from the external auditory canal to the inferior orbit) and eyes focused on a point at eye level, legs parallel, and heels, calves, buttocks, scapulae, and occiput ( back of the head) pressed against the stadiometer or wall. Weight was measured using a Welmy^®^ digital anthropometric scale with a capacity of 200 kg and an accuracy of 100 g. The patient was positioned in the center of the scale, barefoot, upright, arms extended along the body, with the feet together and in such a way that the weight was distributed symmetrically, avoiding support more firmly on one of the legs and with the gaze fixed. on a point on the straight line.

Based on measured weight and height measurements, BMI was calculated according to the formula BMI = weight (kg)/height (m^2^) and classified according to the World Health Organization^(22)^ as underweight (≤18.5 kg/m2), normal weight (between 18.6 and 24.9 kg/m2), overweight (between 25 and 29.9 kg/m2) and obese (≥30 kg/m2). The different BMI categories were grouped for better analysis, initially BMI ≤ 24.9 kg/m2, BMI between 25 and 29.9 kg/m2 and BMI ≥30 kg/m2.

Waist circumference (WC) was measured in an upright posture with the abdomen relaxed, arms outstretched, legs parallel and slightly apart, waist uncovered, and breathing normal. The last rib was located and marked during inhalation. The iliac crest is located and marked. The midpoint between the two anatomical points was calculated and marked where the zero point of the tape was positioned, and the tape was passed around the waist without being tight or loose and at the same level in all parts of the waist. The patient was asked to inhale and then exhale completely. The measurement was recorded at the end of the breath.

Laboratory samples were collected during the women’s visit to the research center. Blood glucose and lipid profile (triglycerides, total cholesterol, high-density lipoprotein (HDL) cholesterol) were measured with an automated biochemist (Weiner, Rosario, Argentina).

The National Cholesterol Education Program Adult Treatment Panel criteria III (NCEP-ATP III), revised by the American Heart Association and the National Heart, Lung, and Blood Institute (AHA /NHLBI),^(5)^ were used to diagnose the metabolic syndrome. The choice of this criterion was based on the one most used in Brazil and recommended by the Brazilian Society of Cardiology.

Thus, a metabolic syndrome is present when three of the five criteria below are met:

- Central obesity - waist circumference of more than 88 cm (personally measured during the collection of anthropometric measurements);
- Hypertension - systolic blood pressure ≥130 and/or diastolic blood pressure ≥ 85 mmHg (measured in person at the time of anthropometric data collection) or use of antihypertensive medication or previous diagnosis of arterial hypertension;
- Altered blood glucose (blood glucose ≥110 mg/dl) or diagnosis of diabetes or taking medication to treat diabetes mellitus;
- Triglycerides ≥150 mg/dl or previous diagnosis of dyslipidemia or use of hypolipidemic agents;
- HDL cholesterol ≤ 50 mg/dl or previous diagnosis of dyslipidemia or use of lipid-lowering agents.

For risk factor analysis, women were divided into two groups: one with metabolic syndrome and one without metabolic syndrome.

To search for factors associated with metabolic syndrome, univariate analysis was performed using the covariates described above. Binomial logistic multiple regression was performed, and the adjusted relative risk (RR) and 95% confidence interval (95% CI) were calculated. Univariate analysis using the covariates described was applied to identify predictors of metabolic symdrome. All variables with P < 0.10 in the univariate analysis were included in a multiple logistic regression model.

After analysis of factors associated with metabolic syndrome in all women in the study, patients who had never been pregnant were excluded. The aim was to analyze the reproductive and obstetric characteristics associated with metabolic syndrome in women with a previous pregnancy. The same statistical strategy was used for this analysis, with unadjusted and adjusted RR obtained by binomial logistic regression. All variables with P < 0.10 were included in the multivariate model.

Data from individual patients were entered into Excel spreadsheets to create a database. The SAS 9.3 program (SAS Institute Inc, Cary, NC, USA) was used for all statistical analyzes. A significance level of 5% (p < 0.05) was used. Missing data were excluded from the analysis.

This research was conducted in accordance with the Declaration of Helsinki and approved by the Ethics Committee of the Faculty of Medicine of Ribeirão Preto, University of São Paulo 1.282.710 – CAAE 45485915.7.0000.5440. All phases of this cohort were submitted to and approved by the Research Ethics Committee.

## Results

The study included 929 women who were used for data collection. Women who were pregnant at the time of the study (n=2), women who did not undergo anthropometric data collection (n=6), and women who did not answer the obstetric history questionnaire (n=5) were excluded. Subsequently, 916 women were evaluated.

Most of them (80.2%) were white, had a paid job (90%), lived with a partner (67.2%), had more than 12 years of schooling (45.9%), belonged to socioeconomic class A/B (68%), had ever been pregnant (77.7%), were nonsmokers (88.8%), did not drink alcohol (78.4%), and did not use illicit drugs (96.6%) (Table 1). The prevalence of metabolic syndrome was 31.2%. The prevalence of clinical comorbidities was 11.2% for diabetes mellitus, 19.5% for systemic arterial hypertension, 18.3% for dyslipidemia, 34.7% for overweight, and 33.7% for obesity (Table 1).

**Table 1:** Sociodemographic and clinical characteristics of women in the Ribeirão Preto cohort 1978/79 (n=916)

Variables		n(%)
Race/ethnicity	White	735(80.2)
	Other (black, brown, yellow)	181(19.8)
Paid work	Yes	720(90.0)
	No	80(10.0)
Marital status	With a companion	614(67.2)
	Without a companion	300(32.8)
Educational level(years of schooling)	≤ 8	110(12.1)
	9 – 11	382(42.0)
	≥ 12	417(45.9)
Socioeconomic class	A/B	598(68.0)
	C	262(29.7)
	D/E	20(2.3)
Previous pregnancy	Yes	712(77.7)
	No	202(22.3)
BMI classification	Low weight or adequate	289(31.6)
	Overweight	318(34.7)
	Obesity	309(33.7)
Diabetes mellitus	Yes	102(11.2)
	No	811(88.8)
Dyslipidemia	Yes	167(18.3)
	No	744(81.7)
Arterial hypertension	Yes	178(19.5)
	No	734(80.5)
Metabolic syndrome	Yes	286(31.2)
	No	630(68.8)
Smoking	Yes	102(11.2)
	No	812(88.8)
Alcohol misuse	Yes	171(21.6)
	No	620(78.4)
Illicit drug use	Yes	31(3.4)
	No	385(96.6)

The diagnosis of metabolic syndrome was made using clinical variables in conjunction with anthropometric data, blood pressure measurement, and laboratory test results (blood glucose and lipidogram). Table 2 shows the percentage of women in whom these variables were altered within the diagnostic criteria for MetS.

**Table 2:** Waist circumference, blood pressure measurement, and results of laboratory tests for fasting glucose, triglycerides, and HDL cholesterol in women of the 1978/79 cohort

Variables		n(%)
Waist circumference > 88 cm		404(44.1)
Blood pressure		
	Systolic ≥130 mmHg	148(16.1)
	Diastolic ≥ 85 mmHg	174(19.0)
Fasting glucose ≥ 110 mg/dl		107(11.7)
Triglycerides ≥150 mg/dl		219(23.9)
HDL cholesterol ≤ 50 mg/dl		516(56.33)

Table 3 shows the binomial logistic multiple regression analysis performed to determine the predictors for MetS. After multivariate analysis, the risk factors for metabolic syndrome remained lack of paid work (RR 1.49; 95% CI 1.14-1.95), marital status without a partner (RR 1.33; 95% CI 1.03-1.72), and less than 8 years of schooling (RR 1.72; 95%CI 1.23-2.41) and 8 to 12 years of schooling (RR 1.37; 95%CI 1.06-1.76) compared with more than 12 years of schooling (educational level). The other covariates were not associated with MetS. There was no collinearity between the variables included in the multiple regression models.

**Table 3:** Sociodemographic and reproductive factors associated with metabolic syndrome in women of the 1978/79 cohort

Variables		Metabolic Syndrome		Unadjusted RR* (95% CI)	Adjusted RR** (95% CI)
		Yes (n=286) n(%)	No (n=630) n(%)		
Race/ethnicity					
	White	224(78.3)	511(81.1)	1.00 (reference)	
	Other	62(21.7)	119(18.9)	1.12 (0.89-1.41)	
Paid work					
	Yes	208(85.2)	512(81.1)	1.00 (reference)	1.00 (reference)
	No	36(14.8)	44(7.9)	1.55 (1.19-2.03)	1.49 (1.14-1.95)
Marital status					
	With a partner	206(72.3)	408(64.9)	1.00 (reference)	1.00 (reference)
	Without a partner	79(27.7)	221(35.1)	1.27 (1.02-1.58)	1.33 (1.03-1.72)
Educational level					
	≤ 8 years	52(18.3)	58(9.3)	1.95 (1.50-2.53)	1.72 (1.23-2.41)
	9 – 11 years	131(46.1)	251(40.1)	1.41 (1.13-1.76)	1.37 (1.06-1.76)
	≥ 12 years	101(35.6)	316(50.6)	1.00 (reference)	1.00 (reference)
Socioeconomic class					
	A/B	171(62.2)	427(70.6)	1.00 (reference)	1.00 (reference)
	C	97(35.3)	165(27.3)	1.29 (1.05-1.58)	1.15 (0.91-1.46)
	D/E	7(2.5)	13(2.1)	1.22 (0.66-2.25)	1.01 (0.42-2.42)
Previous pregnancy					
	Yes	227(79.6)	483(76.8)	1.12 (0.88-1.43)	
	No	58(20.3)	146(23.2)	1.00 (reference)	
Number of pregnancies					
	0	58(20.3)	146(23.2)	1.00 (reference)	1.00 (reference)
	1	61(21.4)	187(29.7)	0.86 (0.63-1.17)	0.77 (0.55-1.06)
	≥2	166(58.3)	296(47.1)	1.26 (0.98-1.62)	0.90 (0.68-1.20)
Smoking					
	Yes	37(13)	65(10.3)	1.18 (0.90-1.56)	
	No	248(87)	564(84.7)	1.00 (reference)	
Alcohol misuse					
	Yes	49(20.3)	122(22.2)	0.92 (0.70-1.19)	
	No	193(79.7)	427(77.8)	1.00 (reference)	
Illicit drug use					
	Yes	12(4.2)	19(3.0)	1.25 (0.79-1.96)	
	No	274(95.8)	611(97)	1.00 (reference)	

* Simple binomial logistic regression;

** Multiple binomial logistic regression;

RR - relative risk; 95% CI - 95% confidence interval; Variables included in the multivariate model - paid work, marital status, educational level, socioeconomic class, and number of pregnancies

After analyzing the factors that were associated with MetS in all women in the study, patients who had never been pregnant were excluded. After multivariate analysis, only teenage pregnancy (before 20 years of age) continued to be associated with MetS in women with a previous pregnancy compared with age at first pregnancy over 30 years (RR 2.00, 95% CI 1.45-2.77). The other covariates were not associated with MetS. Table 4 shows the unadjusted and adjusted RR of the covariates studied. There was no collinearity between the variables included in the multiple regression models.

**Table 4:** Reproductive and obstetric factors associated with metabolic syndrome in women of the 1978/79 Ribeirão Preto cohort who had at least one pregnancy

Variables		Metabolic Syndrome		Unadjusted RR* (95% CI)	Adjusted RR** (95% CI)
		Yes (n=228) n(%)	No (n=484) n(%)		
Age at first pregnancy					
	< 20 years	90(41.1)	114(24.9)	2.00 (1.44-2.76)	2.00 (1.45-2.77)
	20 - 29 years	92(42.0)	213(46.5)	1.36 (0.98-1.90)	1.37 (0.98-1.91)
	≥ 30 years	37(16.9)	131(28.6)	1.00 (reference)	1.00 (reference)
Previous abortion					
	Yes	56(24.6)	128(26.5)	1.00 (reference)	
	No	172(75.4)	356(73.5)	1.07 (0.83-1.37)	
Parity*					
	0	8(3.5)	26(5.4)	1.00 (reference)	
	1	72(31.6)	205(42.3)	1.10 (0.58-2.09)	
	≥ 2	148(64.9)	253(52.3)	1.56 (0.84-2.91)	
Previous cesarean section					
	Yes	157(68.9)	323(66.7)	1,06 (0.84-1.34)	
	No	71(31.1)	161(33.3)	1.00 (reference)	
Breastfeeding (> 50%)					
	Yes	200(87.7)	421(87)	1.00 (reference)	1.00 (reference)
	No	28(12.3)	63(13)	0.95 (0.68-1.32)	1.07 (0.74-1.56)

* Simple binomial logistic regression;

** Multiple binomial logistic regression;

RR - relative risk; 95% CI - 95% confidence interval; Variables included in the multivariate model: age at first pregnancy and breastfeeding

## Discussion

The population consisted of women aged 37 to 39 years from a cohort study conducted in the city of Ribeirão Preto. Participants were recruited by telephone, through advertisements in the media and social networks, and by searching a digital environment, strategies that may not have reached some groups. Only 1,775 of 6,973 women were then available for data collection. This sample represents 25% of the original sample, a loss of 75% of the individuals. This loss may result in a significant bias in the results of this study.

The prevalence of metabolic syndrome was 31.2%. The prevalence of clinical comorbidities was 11.2% for diabetes mellitus, 19.5% for systemic arterial hypertension, 18.3% for dyslipidemia, 34.7% for overweight and 33.7% for obesity metabolic syndrome.

Women were divided into two groups according to the presence or absence of metabolic syndrome to identify factors associated with this disorder. The associated sociodemographic factors were low educational level (less than 8 years of schooling and 8 to 12 years of schooling compared with more than 12 years), lack of a paid job, and lack of a partner, and the only factor associated with reproductive health was teenage pregnancy (< 20 years).

Considering that almost a third of the population of women in this study have a diagnosis of metabolic syndrome is a concern and reflects the need for attention to the problem. The high rates of MetS in the studied population suggest that the sample is susceptible to other metabolic disorders and consequently to increased cardiovascular risk.^(23)^

Comparing our incidence data with the literature is an effort, since the articles are very heterogeneous.

Literature data show that the occurrence of MetS coincides with that of obesity and T2DM. Approximately 85% of patients with T2DM also have MetS and are therefore at increased risk for CVD.^(24)^ In 2017, about 12.2% of the US adult population had T2DM. About a quarter of them were unaware of the disease. Not surprisingly, the prevalence of MetS was three times higher and accounted for about one-third of the US adult population. Fortunately, the National Health and Nutrition Examination Survey (NHANES) has released recent data showing that the number of people affected is declining: 24% of men and 22% of women.^(24)^

Few Brazilian studies aimed to estimate the prevalence of metabolic syndrome and identify associated factors, especially in the female population.

A Brazilian study, analysis of a representative sample (n = 59,402) (2013 National Health Survey) revealed a prevalence of metabolic syndrome of 7.5% in men and 10.3% in women.^(25)^ Another study, a systematic review, showed that the overall pooled prevalence of MetS in the Brazilian general population was 33%, with a large heterogeneity. When broken down by sex, the prevalence was 26% in men and 38% in women. Prevalence in the different habitats was 34% in urban areas, 15% in rural areas, 28% in Quilombola, and 37% in the indigenous population. Across regions, prevalence was 37% in the South, 30% in the Southeast, 38% in the North, 31% in the Northeast, and 39% in the Midwest. The pooled prevalence of MetS with age was < 45 years: 43% and ≥ 45 years: 42%, and prevalence by year of study implementation was 31% in 2015–2019, 35% in 2010–2014, and 28% in 2005–2009.^(26)^

A cross-sectional study selected 581 women (aged 35-65 y) from among those enrolled in a family health program in the city of Pindamonhangaba, Brazil. Metabolic syndrome was identified in accordance with the definition of the National Cholesterol Education Program Adult Treatment Panel III. The prevalence of metabolic syndrome was 42.2% (95% CI, 38.1-46.2). The following factors were associated with metabolic syndrome: the 45- to 54-year age group (prevalence ratio, 1.54; 95% CI, 1.08-2.01), the 55- to 65-year age group (prevalence ratio, 3.51; 95% CI, 1.49-3.10), hyperuricemia (prevalence ratio, 2.95; 95% CI, 1.15-1.86), and sleep apnea risk (prevalence ratio, 2.41; 95% CI, 1.16-1.82). The authors found an inverse association between metabolic syndrome and having had more than 5 years of schooling (prevalence ratio, 0.65; 95% CI, 0.65-1.04).^(27)^

A study from Amazonas, Brazil aimed to estimate the prevalence of the individual and general components of metabolic syndrome in adults and older adults and identify the independent predictors of metabolic syndrome. The overall prevalence of metabolic syndrome was 47.5%. Advanced age, being female, having a higher body mass index, and a having lower educational level independently increased the odds of metabolic syndrome.^(28)^

The analysis of data from this study had some limitations. The prevalence determined in the present study may have been influenced by some limitations of this cohort follow-up study, including the recruitment process, the number of women from the original cohort who participated in data collection, and the fact that the data were from a single city.

This percentage of women who were not followed up may have introduced some bias into the study by potentially determining a more common profile in the study. For example, women with more comorbidities might be more interested in health studies than women without chronic conditions, which would increase the prevalence of obesity in the sample.

The characteristics of the women who participated in the 2016/17 assessment made the population more homogeneous. Most of these women were white, had more than eight years of schooling, lived with a partner, held paid jobs, and belonged to a higher socioeconomic class; these conditions may also have introduced some bias into the study. The fact that the study captured a rather homogeneous and biased population, perhaps because of the greater demand for participation in the study by women with chronic conditions, the main outcome of the study, may have hindered statistical analysis. For example, this difficulty may have limited the association of obesity and prior pregnancy. In addition, whether the data were from a single Brazilian city may limit the generalizability of the present results.

In the case of women with higher levels of education, possible explanations include greater social pressure and better access to weight control and weight loss programs, whether or not they are healthy.^(29,30)^ On the other hand, women with low levels of education may have difficulty making better food choices and have limited access to weight loss programs. Among teenage mothers, there are both sociodemographic and physiologic risk factors for obesity and metabolic symdrome. Sociodemographic risk factors include black race/ethnicity, poverty, and low educational attainment.^(31,32)^ Physiological risk factors are higher gestational weight gain and greater postpartum weight retention than that observed in adults.^(33)^

In contrast to expectations, this study did not find an association between other reproductive factors and metabolic syndrome. Pregnancy and postpartum are critical periods for the development of overweight and obesity; however, although the relationship between maternal pregnancy weight and the risk of becoming obese has been the focus of studies in recent years,^(34,35)^ the level of evidence is still dubious. Studies like the present one rely on the physiology of gestational weight gain in part at the expense of body fat accumulation during pregnancy in an attempt to show the association between previous pregnancies and obesity.^(36,37)^

One major strength of this study is its cohort design. Birth cohort studies have been a top priority on the research and technology agenda of developed countries.^(38)^ The assessment of a group of live births over a given period allows to monitor the health of these individuals throughout their lives.^(38)^

In summary, the present results are in line with the global scenario of metabolic syndrome being a highly prevalent disease in adults. Primary prevention strategies are necessary, and attention must be paid to women with low educational level, no paid work, without a partner and to pregnant adolescents. During prenatal care of teenagers, interventions that promote appropriate weight gain are vital to prevent postpartum weight retention because excess gestational weight gain is a strong predictor of maternal overweight and obesity after pregnancy.^(32)^

## Conclusion

Metabolic syndrome in a population of women in the fourth decade of life was associated with lack of employment, lack of a partner, low educational level, and teenage pregnancy.
